# Genome-wide analysis of *E. coli* cell-gene interactions

**DOI:** 10.1186/s12918-017-0494-1

**Published:** 2017-11-23

**Authors:** S. Cardinale, G. Cambray

**Affiliations:** 1 Department of Bioengineering, University of California-Berkeley, Berkeley, CA 94720 USA; 2 Present Address: Technical University of Denmark, Novo Nordisk Foundation Center for Biosustainability, Building 220, 2800 Kgs. Lyngby, DK Denmark; 3 California Institute for Quantitative Biosciences, University of California-Berkeley, Berkeley, CA 94720 USA; 4 DGIMI, INRA, University of Montpellier, Montpellier, France

**Keywords:** Synthetic gene expression, Cell growth, Cellular systems, Positive feedback, KEIO gene knockouts

## Abstract

**Background:**

The pursuit of standardization and reliability in synthetic biology has achieved, in recent years, a number of advances in the design of more predictable genetic parts for biological circuits. However, even with the development of high-throughput screening methods and whole-cell models, it is still not possible to predict reliably how a synthetic genetic construct interacts with all cellular endogenous systems. This study presents a genome-wide analysis of how the expression of synthetic genes is affected by systematic perturbations of cellular functions. We found that most perturbations modulate expression indirectly through an effect on cell size, putting forward the existence of a generic Size-Expression interaction in the model prokaryote *Escherichia coli*.

**Results:**

The Size-Expression interaction was quantified by inserting a dual fluorescent reporter gene construct into each of the 3822 single-gene deletion strains comprised in the KEIO collection. Cellular size was measured for single cells via flow cytometry. Regression analyses were used to discriminate between expression-specific and gene-specific effects. Functions of the deleted genes broadly mapped onto three systems with distinct primary influence on the Size-Expression map. Perturbations in the Division and Biosynthesis (DB) system led to a large-cell and high-expression phenotype. In contrast, disruptions of the Membrane and Motility (MM) system caused small-cell and low-expression phenotypes. The Energy, Protein synthesis and Ribosome (EPR) system was predominantly associated with smaller cells and positive feedback on ribosome function.

**Conclusions:**

Feedback between cell growth and gene expression is widespread across cell systems. Even though most gene disruptions proximally affect one component of the Size-Expression interaction, the effect therefore ultimately propagates to both. More specifically, we describe the dual impact of growth on cell size and gene expression through cell division and ribosomal content. Finally, we elucidate aspects of the tight control between swarming, gene expression and cell growth. This work provides foundations for a systematic understanding of feedbacks between genetic and physiological systems.

**Electronic supplementary material:**

The online version of this article (10.1186/s12918-017-0494-1) contains supplementary material, which is available to authorized users.

## Background

Synthetic biology seeks to enable the design of novel cell functions of increasing complexity through standardization of biological engineering. This goal critically depends on the reliability and predictability of individual synthetic biological components and their composition [1, 2]. Genetic constructs designed to accomplish specific functions in the cell are constantly challenged by mutable endogenous interactions, which can quickly render them unstable or non-functional through modification of the host physiology or genetic makeup. To address these issues, tools are being engineered to shield the functions or predict the behavior of synthetic genes in the cell. These include the development of devices to mitigate the influence of changing molecular context [3], design guidelines to improve molecular robustness to evolutionary instability [4] and the application of computational algorithms to achieve parametrically robust circuits [5].

Genome-wide mapping of the gene-to-phenotype relationships has enabled the effective identification of genetic targets to improve complex traits in bacteria, such as tolerance to ethanol [6] or cellulosic hydrolysate and isobutanol [7]. This information, however, does not guarantee the success of engineering heterologous pathways in these strains. To achieve this capability, one would need accurate models of the biochemical networks at the whole cell level [8]. A systematic understanding of the relationships and feedbacks linking cell function and gene expression is required to build such models.

In this work, we investigated the interaction between cell function and synthetic gene expression in knockouts of all non-essential genes in the *E. coli* genome. Specifically, we mapped the global effect of single-gene deletions using cell size as an integral proxy of cellular biogenesis, and specific effects on individual synthetic genes via a dual-fluorescence genetic construct. In some instances, these two measurements can be intricately related. The global effect may associate the ability of the cell to grow and the cellular amount of a synthetic genetic component through growth feedback, which have been described mathematically [9]. Alternatively, disruption of a particular cell function may cause more restricted effect on the output of particular synthetic genes without impacting cell size or growth. To investigate such restricted effect, we used a genetic construct with two fluorescent reporter genes under the control of identical promoter and 5′ UTR sequence, as we described previously [10].

We mapped the phenotypic patterns of the size-expression interaction to three major systems in the cell: Membrane and Motility **(MM**)**,** ribosome-protein synthesis driven by nutrients (Energy, Protein synthesis and Ribosome, **EPR**), and biosynthetic or cell progression functions (Division and Biosynthesis, **DB**). An impairment of cell division determined larger cells and the lower dilution rate of the cell content indirectly resulted in higher cellular reporter concentrations (growth feedback). In contrast, defective motility yielded smaller cells and reduced gene expression, both key aspects of the highly regulated switch between swarming and biofilm formation that is linked to central carbon metabolism. Finally, protein synthesis and folding functions directly and differentially affected synthetic reporter expression with secondary implications on cell size, especially when the disruption concerned structural components of the ribosome.

## Methods

### Strains, plasmids and media

Single-gene knockout strains were obtained from the KEIO collection (National BioResource Project - SHIGEN) [11] and wild-type laboratory strains of *E. coli* from the Joint Bio-Energy Institute (JBEI, Emeryville-CA). The construction of pEZ8–123 synthetic genetic probe has been previously described [12]. To introduce this reporter plasmid in the strain library, cells were cultivated in LB media supplemented with Kanamycin at a concentration of 50 μg/ml and subjected to chemical (CaCl_2_) transformation in batch (96 per KEIO plate). For flow cytometry and all further assays, cells were grown in Neidhardt’s MOPS-based Rich defined medium (Teknova), supplemented with 0.5% glucose and antibiotics Ampicillin or Kanamycin (50–70 μg/ml).

### Flow cytometry

24–36 strains (2–3 rows for each 96 well plate) were grown concurrently after inoculation in warm MOPS rich medium supplemented with 0.5% glucose from overnight cultures grown in the same media (1:80 dilution). Cells were grown for exactly 1h15min, in a shaker incubator at 37°C. Optical densities at 600 nm were measured with a microtiter plate reader to identify cultures in mid-exponential phase. These cultures were diluted 1:200 in PBS + 100 μg/ml G418 to inhibit protein synthesis, and single-cell readings were acquired with a Guava Flow Cytometer (Merck).

### Statistical and computational analysis

The R software and appropriate Bioconductor packages were used to develop custom scripts for data and subsequent statistical analysis. Please refer to supporting information for a detailed description of methodologies and functions.

## Results

### Quantification of cell size and reporter expression across the KEIO collection

**Fig. 1:**
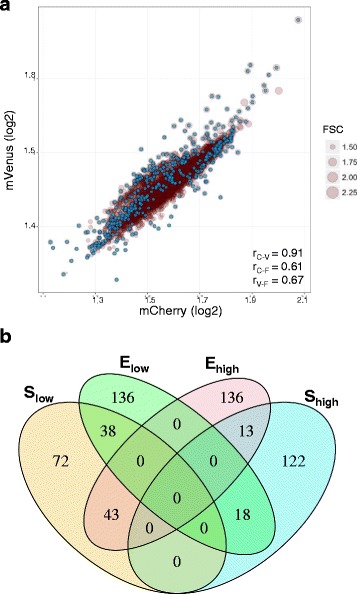
**a** Measurement of synthetic gene expression and cell size are correlated. Scatterplot of the population mean expression of mVenus expression as a function of mCherry expression, as derived from single cell measurements. Point sizes are proportional to the cell volume (FSC). Cyan points highlight significant knockouts after FSC regression (r: Pearson correlation coefficients). **b** Venn diagram of the overlap between strains with **S**_**high**_, **S**_**low**_, **E**_**high**_ or **E**_**low**_phenotype (top and bottom 5% quantile of the respective distributions)

The goal of this study is to comprehensively characterize how loss of gene function impacts two key optimization parameters in synthetic biology and metabolic engineering: cell biomass and heterologous gene expression. To do this, we quantified the effect of every single deletion of non-essential genes in *E. coli* on cell size and the expression levels of two constitutively expressed synthetic reporter genes. A genetic probe containing the mVenus and mCherry genes expressed from identical promoter-5′-UTR sequences [12] was transformed into each of the 3822 gene knockout strains of the KEIO collection [11] and into two independent cultures of the wild-type parent strain (*E. coli* BW25113) (Fig. 1a). Each of the 3824 strains carrying the probe was grown from a mixture of 2–3 single colonies picked from agar plates to mitigate potential colony-to-colony variability.

Single-cell measurements of mVenus and mCherry fluorescence, along with cellular physical parameters, were acquired with a flow cytometer at the mid-log phase of growth. Fully replicating these measurements on the whole library was not practical. To estimate the experimental error associated with plate-wise measurement, we performed replicate measurements of 180 strains picked from three different plates on 4 different days. Measurement errors for both mVenus and mCherry were approximately one order of magnitude smaller than the variance measured across all KEIO strains for these variables (Additional file 1). This readily demonstrates substantial impact of the gene deletions on heterologous expression. Although forward scattered light (abbreviated FSC) can be effectively used to measure microbial cell size in flow cytometry [12], it does not necessarily scale linearly with particle sizes on all instruments [13, 14]. We used beads to verify that this was the case in our instrument within the size range typical of an *E. coli* cell (~2μm, Additional file 1: Fig. S1). We therefore used FSC as a proxy for cellular size (**S**).

As we followed the original layout of the Keio collection [11], our strains are not distributed randomly amongst plates. In fact, genes with similar functions were occasionally grouped together in the same plate. For example, many genes encoding chemotactic and flagellar proteins are clustered in plate #45 (Additional file 1: Fig. S2). This non-random arraying of strains could have introduced bias in our measurements. However, we did not observe significant plate-specific shifts of median fluorescence in relation to the whole dataset distribution (Additional file 1: Figs. S3-S6). Re-arraying of 180 strains and additional analysis further confirmed this conclusion (see Additional file 1). Therefore, to avoid the risk of introducing processing bias in the dataset, no further data normalization was performed.

The average fluorescence of mVenus and mCherry varied approximately four-fold and was strongly correlated across the 3.824 strains (*r* = 0.90, Fig. 1a) and with **S** (correlation 0.67 and 0.61 for mVenus and mCherry, respectively) (Fig. 1a). A change in cell size could indirectly affect heterologous gene expression in cases in which proteins are not sufficiently split between daughter cells during cell division (growth feedback) [9]. This scenario was supported by the observed positive correlation between FSC and fluorescence output (Fig. 1a). To quantify the specific effects of gene knockout on heterologous gene expression we needed to account for the influence of cell size variations **(S).** Measurements of mCherry and mVenus fluorescence were regressed against **S.** Pairwise averages of resulting residuals (mC^reg^ and mV^reg^) were used as **S**-normalized measure of heterologous gene expression (**E**). To quantify the differential effect of knockouts on the individual reporter genes, we used the residuals obtained upon regressing mC^reg^ and mV^reg^ against **E**. This regression yielded identical sets of absolute values (residuals) that remained highly correlated with **E** (*r* = 0.94–0.98) and were used as proxy for gene-specific effects (**G**_**spec**_) (strains with significant difference between mCherry and mVenus fluorescence) (Additional file 1).

### Most KEIO knockouts show a single-feature phenotype

To ease the analysis of associations between variables, we binned strains into groups of extreme phenotypic values (top and bottom 5% quantiles giving respectively **S**_**high**_ / **S**_**low**_ and **E**_**high**_ / **E**_**low**_). These extreme values were homogenously distributed across the dataset (Fig. 1a, cyan dots), showing that the regression procedure did not introduce systematic biases. About 192 genes showed an extreme **S** or **E** value, whereas the number of genes with a **G**_**spec**_ phenotype was 384.

Amongst the set of unique 578 strains thus selected, 81% presented exclusively one extreme **E** or **S** phenotype. A **S**_**high**_ phenotype was very rarely combined with extreme expression (**E**_**high**_ or **E**_**low**_, ~2% each). In contrast, the **S**_**low**_ phenotype was significantly associated with **E** phenotypes (~7% each, *p* < 10^−4^, Fig. 1b, calculated by bootstrap against random occurrence). Extreme **E** and **G**_**spec**_ phenotypes were found in combination in only 14–19% of strains**.** Notably, 33% **(**53/159) of genes with a **S**_**low**_ shift also had a **G**_**spec**_ phenotype compared with only 7.5% of those with a **S**_**high**_ phenotype. This result suggests that deletions leading to smaller cells have larger chance to disrupt the balance in the expression of two synthetic genes than genetic perturbations increasing cell size (Fig. 1b).

We assessed the presence of functional enrichments in strains with a single **S**, **E** or **G**_**spec**_ phenotype using DAVID Bioinformatics Resources [15]. The **S**_**low**_ and **S**_**high**_ categories did not present functional enrichment after Bonferroni correction for multiple-hypothesis testing. The **E**_**low**_ group was significantly enriched in knockouts of genes involved in flagella assembly (GO:0044780 Bonferroni corrected, *p* < 0.05). Strains with a **G**_**spec**_ phenotype corresponded to a diverse range of cell functions including transcription factors and enzymes involved in central carbon metabolism, with a significant enrichment in amino acid biosynthesis related genes (KEGG pathway, *p* < 10^−2^). Apart from a number of genes involved in purine nucleotide biosynthesis, the **E** and **G**_**spec**_ phenotypes did not share substantial sets of cellular functions.

### Detailed functional analysis of the size-expression relationship

**Fig. 2:**
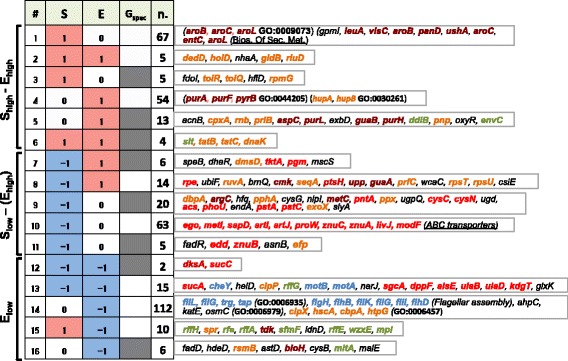
Distribution of genes in 16 phenotypic patterns with significantly (>2 st. dev.) increased (+1/pink) or decreased (−1/cyan) cell size (**S**), global gene expression (**E**) and gene-specific effects (**G**_**spec**_ **)**. For each combination of **S**, **E** and **G**_**spec**_ patterns either the individual extreme genes (for <20 genes) or the functional enrichments (DAVID) of Gene Ontology classes (bold/GO) or KEGG pathways (underscored) is listed. (Text color: brown = amino acids and nucleotides biosynthesis; orange = important to cell growth; red = nutrient uptake and catabolic reactions; cyan = motility and chemotaxis function; dark green = cell membrane structural component) (n.: number of genes; Grey cells: significant **G**_**spec**_ phenotype)

To gain a better understanding of the role of different cell functions on the relationship between cell size and gene expression, KEIO knockouts populating all pairwise combinations of extreme phenotypes were investigated. To specifically investigate strong **S – E** associations, we only considered combinations where an extreme Z-score (St. Dev.-fold from the mean) for one variable was combined with a near-zero value (−0.5 to 0.5 range) for the other (compare one-feature categories in Fig. 2 and Fig. 1b). The presence of a **G**_**spec**_ effect, which quantifies expression imbalance between the two reporter genes, was assessed in each of the observed **S - E** patterns. This method defined 16 phenotypic combinations corresponding to 401 strains. Combinations were arranged in a matrix with **S**_**high**_ and **E**_**low**_ placed at the top and bottom, respectively (Fig. 2). This arrangement exposed that most genes are characterized by a similar up/down shift in the **S** and **E** features. Only 30 (7.5%) of these strains presented a mixed phenotype (for example **S**_**down**_ **– E**_**high**_). All phenotypes with >20 members were assessed for functional enrichment, while gene lists were reported for smaller groups.

Gene disruptions that severely affected both **S** and **E** phenotypes impaired major cellular functions. Only impairments in amino acid biosynthesis, and particularly in aromatic amino acids, led to an exclusive **S**_**high**_ phenotype (Bonferroni corrected *p* < 10^−1^) (Fig. 2 group #1 brown genes). Genes with a significant **S**_**high**_ or **E**_**high**_ phenotype, either alone or in combination, were often related to cellular housekeeping functions (Fig. 2, Groups #2–8 orange genes). Intriguingly, impairing nucleotide biosynthesis primarily results in **E**_**high**_ phenotype, either associated or not with a **S** or **G**_**spec**_ effect (Fig. 2, Groups #4 and #5, brown genes). Altogether, these data show that gene disruptions in amino acid and nucleotide biosynthesis pathways trigger distinct phenotypic increases in cell size and generic gene expression, respectively.

Many knockouts with a **S**_**low**_ phenotype involved nutrient and metal ion uptake, including phosphate (*pstA*, *pstC*), sulfur (*cysC*, *cysN*) and zinc (*ZnuA*, *ZnuB*) (Fig. 2 – groups #8–10 red genes. The combined **S**_**low**_- **E**_**low**_ pattern was populated with strains associated with carbohydrate catabolism (*sucA*, *sucC*) and a critical regulator of stationary phase onset (*dksA*). Knockouts of four major cellular chaperones (Gene Ontology class GO:0006457, Bonferroni corrected *p* < 10^−1^) presented an exclusive **E**_**low**_ phenotype, thus indicating that a lack of protein folding function negatively affects heterologous gene expression (Fig. 2 group 14, discussed below). A majority of knockouts in this phenotypic region (78/105 in groups #9–14, Fig. 2) cause a global and homogenous effect on gene expression as opposed to a specific effect on individual genes (no **G**_**spec**_ effect).

**Fig. 3:**
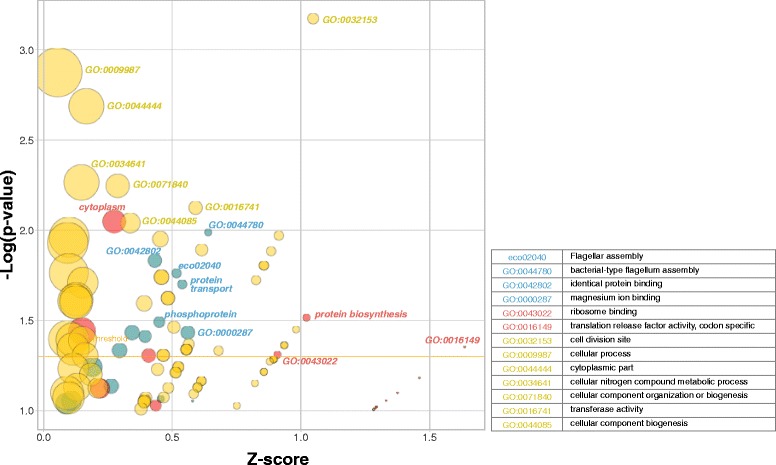
Differential enrichment of GO, KEGG and UP_KEYWORDS functional terms in three groups of genes (Fig. 2): **S**_**high**_ and/or **E**_**high**_ phenotype (Groups #1–6, yellow circles), predominant **S**_**low**_ phenotype (Groups #7–11, red circles) and predominant **E**_**low**_ phenotype (Groups #12–16, blue circles). Axes represent enrichment *p*-value (log_10_ transform, *y*-axis) and the enrichment’s z-score (*x*-axis). Functional terms selected for differential enrichment (legend) have very significant score (>2) or p-value (<0.1), or pass a Log_10_(p) ≤ 1.3 and z ≥ 0.5 combined threshold

The **S**_**low**_ - **E**_**low**_ phenotype was also associated with several knockouts of genes involved in bacterial chemotaxis (*cheY*, *motA*, *motB*), while exclusive **E**_**low**_ phenotypes are linked to disruptions in flagellum assembly (GO: 0006935 and KEGG pathway flagellar biosynthesis) (Fig. 2 - Group #14 and Fig. 3, Bonferroni corrected *p* < 0.05). These observations show that cell motility is implicated in cell-wide changes of gene expression in *E. coli* (discussed below). Disruptions in the ‘Enterobacterial Common Antigen Biosynthetic Process’ (GO:0009246) also showed an **E**_**low**_ phenotype – but combined with a **S**_**high**_ shift, in contrast to chemotaxis genes (Fig. 2 – group #15). Inclusion of 13 other members of this ontology group that only showed a mild score further strengthened the association with a **E**_**low**_ phenotype (*p* < 10^−4^, calculated via bootstrapping). Unlike many knockouts of genes involved in cell growth, which predominantly led to **S**_**high**_ - **E**_**high**_ phenotypes (Fig. 2, Groups #2–3), the **S**_**high**_ – **E**_**low**_ response triggered by ECA knockouts suggested the existence of a different underlying mechanism.

### Growth, protein synthesis and motility are distinct systems of the S-E landscape

Our analyses above revealed that the phenotypic impacts of single-gene deletions define a **S-E** landscape with three main regions: jointly higher size and expression (**S**_**high**_ – **E**_**high**_, Fig. 2 Groups #1–6), predominantly reduced size with neutral or increased expression (**S**_**low**_ - **E**_**high**_, Fig. 2 Groups #7–11) and predominantly reduced expression (**E**_**low**_, Fig. 2 – Groups #12–16). To obtain a broader understanding of this landscape, we performed another functional enrichment analysis amongst the strains populating these regions [16]. Functional terms passing both a *p*-value (*p* < 0.1) and a Z-score (z > 0.5) threshold were defined as differentially enriched (Fig. 3) (Bioconductor package CompGO, Additional file 1).

The **S**_**high**_ – **E**_**high**_ region harbored strains deleted of key bacterial growth functions including cell division and important housekeeping cytosolic cellular processes (GO:0009987 and GO:0044444, Cellular Components) (Fig. 3 yellow circles). A detailed inspection of child GO Biological Processes (BP) revealed enrichment for functions involved in iron-sulfur cluster assembly (GO:0016226, *iscA*, *sufC*, *cyaY*, *ygfZ*), mRNA degradation (GO:0006402, *rnr*, *pnp*), chromosome condensation (GO:0030261, *hupAB*) and DNA-templated transcriptional regulators (GO:0006335, *oxyR*, *mfd*). A significant number of KEIO strains associated with the *E. coli* GO class ‘DNA-dependent DNA replication’ (GO:0006261) were also found associated with a **S**_**high**_ – **E**_**high**_ phenotype (Bonferroni corrected *p* < 10^−2^, 5/9 Additional file 1: Fig. S11a-c).

The **S**_**low**_ **- E**_**high**_ region was mainly populated by strains knocked out of cytoplasmic factors involved in protein biosynthesis (GO:0043022 and GO:0016149) (Fig. 3 – red circles). These included structural or functional components of the ribosome and important factors involved in translation (*prfC*, *efp, queA*). A total of twelve ribosome structural genes could be deleted in the KEIO collection. Out of these, 5 showed a **S**_**low**_ – **E**_**high**_ phenotype (*rpsU*, *rpsT*, *rpmJ*, *rpmE*, *rplA, p* = 0.02, calculated by bootstrap, Additional file 1). More generally, 27 KEIO strains deleted for genes with a key role in translation (GO:0006412) showed a strong **S**_**low**_ – **E**_**high**_ pattern (*p* < 10^−2^) (Additional file 1: Fig. S11d). Significantly, 18 of these genes also showed a significant **G**_**spec**_ phenotype (p < 10^−4^). Thus, disruption of non-essential genes involved in translation had a differential effect on the expression of individual heterologous genes.

The **E**_**low**_ region was associated with knockouts of structural flagellar proteins in the analysis above. A broader search confirmed a significant enrichment for GO:0044780 (cell motility) and the KEGG pathway eco02040 (flagellar assembly) (Fig. 3 – blue circles). A comprehensive analysis of genes involved in chemotaxis (GO:0006935, 23 genes) and flagella (GO:0009288, 23 genes) further supported the **E**_**low**_ phenotype (p < 10^−4^ and *p* < 0.005, respectively). More than half (14/23) of the genes in the latter group had also a **S**_**low**_ phenotype (Additional file 1: Fig. S11E and Fig. 2 – Group #13).

## Discussion

**Fig. 4:**
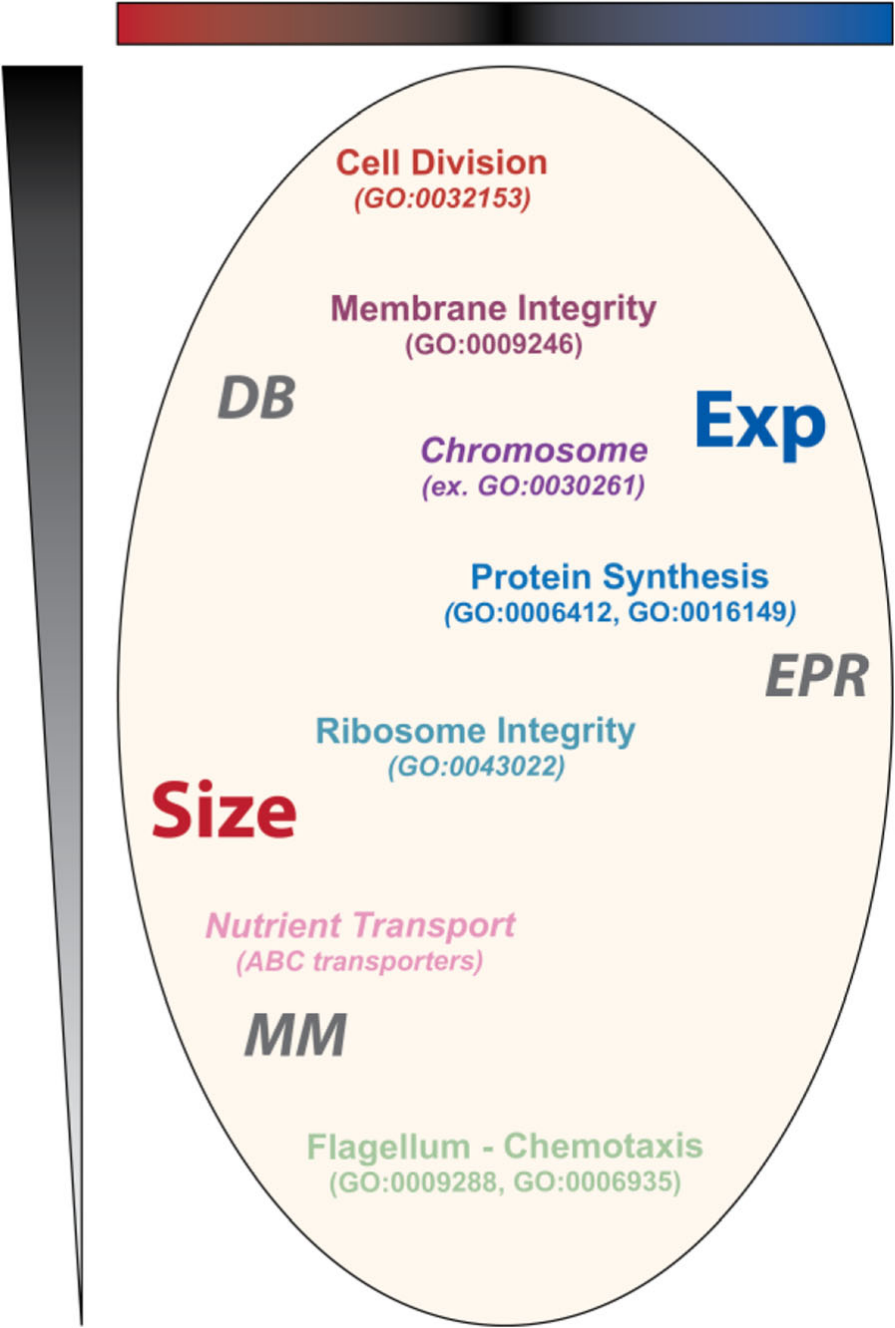
A map of the effect of three major cellular systems **DB** (Division-Biosynthesis), **EPR** (Energy, Protein synthesis and Ribosome) and **MM** (Motility and Membrane) on the interaction between cell Size (red) and synthetic gene Expression (Exp, blue). Cell function disruption can predominantly affect Size, Exp or both (represented by color gradient from dark red to dark blue) by increasing or reducing their value (from top to bottom)

Patterns of phenotypic effect resulting from the disruption of individual genes outline three main functional systems: i) the Division and Biosynthesis (**DB**) system comprises 121 genes with a predominant **S**_**high**_ or **E**_**high**_ phenotype (Fig. 2, groups #1–6; Fig. 4 - top); ii) the Energy, Protein synthesis and Ribosome (**EPR**) system contains 88 genes whose disruption lead to a **S**_**low**_ phenotype (Fig. 2 - groups #9–11; Fig. 4 - middle); and iii) the Membrane and Motility (**MM**) system encompass 127 genes whose absence results in **S**_**low**_ **- E**_**low**_ phenotypes (Fig. 2 - groups #13-14; Fig. 4 - bottom).

Knockout strains linked to the **DB** system involve genes responsible for cell division (GO:0032153) and the biogenesis of ribonucleoproteins and membrane components (GO:0044085 and 0022613). These likely result in cell size increase (**S**_**high**_ **)** because of defects in cell division. The inverse correlation between cellular concentration of a constitutively expressed protein and the rate of growth has been known for several decades [16, 17]. This dependence originates from the growth dependencies of several cellular parameters some of which (transcription) tend to increase protein abundance, and others (dilution rate, cell volume) to decrease it. This relationship could be responsible for the **E**_**high**_ phenotype found in combination with **S**_**high**_ for gene knockouts within the **DB** system (Fig. 4 – top). However, growth rate effects alone are not sufficient to fully describe gene expression output [18], and these alternative factors could underlie the **S**_**low**_ - **E**_**high**_ phenotype observed with disruptions of membrane ECA components.

Disruptions of **EPR** functions primarily cause a **S**_**low**_ phenotype. A weakening of protein synthesis (GO:0006412 and 0016149) or ribosome function (GO:0043022) may trigger a global response similar to that caused by amino acid over-flow, mediated by the alarmone ppGpp. Our data suggest that initially the response would improve cell division or alternatively slow biomass generation [19], resulting in smaller cells. Secondarily, it could dictate an increase in the number of ribosomes to equilibrate nutrient intake, which relies on membrane proteins, with biosynthetic capacity [20, 21] (Fig. 4 – middle), leading to the **E**_**high**_ phenotype observed with some knockout strains of ribosomal or translational proteins (Fig. 2 – Group 8).

The central metabolism is connected to signal transduction via acetyl phosphate. This molecule acts as an important cellular hub connecting nutrient availability, global gene regulation, cell motility, and cell division [22]. For example, serine depletion was shown to result simultaneously in increased motility and reduced cell division rate through acetyl phosphate [23]. Acetyl phosphate levels are thought to control a switch between ‘swarming’ and ‘sticking’ phenotypes, i.e. between motility and biofilm formation [22]. We found that most disruptions in the **MM** involve motility genes and not fimbriae (involved in biofilm formation). The characteristic **S**_**low**_ **- E**_**low**_ phenotype in these strains could arise from a simultaneous reduction in gene expression (via high levels of OmpR-P [24] or global protein acetylation [25]) and cellular biomass accumulation (e.g. growth) (Fig. 4 - bottom).

Most mutations affecting housekeeping cell functions including cell motility, membrane structure, chromosomal DNA replication, repair and homologous recombination, do not differentiate between identically expressed synthetic genes (no **G**_**spec**_ phenotype). Not surprisingly, gene knockouts with differential effects on the two reporter genes were primarily related to protein expression and were associated with functions including ribosome biogenesis (i.e. *dbpA*), translation (*efp*), protein folding (*cpxA*, *dnaK*) and transport (membrane TAT complex) (Fig. 2 – grey cells in **G**_**spec**_ column).

Notwithstanding the caveat that deletion strains are – by definition – not available for essential genes, this study mapped how the removal of every cell function in *E. coli* influences the cell, the synthesis of heterologous genes, or both.

## Conclusions

An important challenge in both metabolic engineering and synthetic biology is to precisely understand how the introduction of engineered or non-native components into a biochemical network influences the behavior of the entire system [26]. For instance, cell biomass, a key optimization parameter in system engineering and biotechnological production, is strongly coupled to heterologous gene expression [27, 28]. A likely consequence is that, though a majority of gene disruptions significantly affect either size or expression, both components are eventually influenced. The data suggest that cellular perturbations could trigger two major global responses: a growth feedback, which determines higher protein or enzyme concentration with larger, non-dividing cells; and a regulatory feedback, where smaller cells could either have higher gene output possibly resulting from up-regulation of ribosome numbers, or lower gene output as consequence of nutritional de-regulation during lack of motility.

The systematic analysis of cellular context of synthetic gene expression given here may facilitate metabolic engineering workflows and systems-level modeling of this model prokaryote, which serves as a key industrial workhorse organism.

## Additional file

Additional file 1: Additional information on methodology, statistical and computational analysis, supplemental figures. (PDF 6.66 mb)

## Abbreviations

DNA: DeoxyriboNucleic acid
RNA: RiboNucleic acid
UTR: UnTranslated Region
