# Supplementary material for: Genome-wide analysis of E. coli cell-gene interactions
Source: BMC Syst Biol. 2017 Nov 23;11:112. doi: 10.1186/s12918-017-0494-1 (PMC5701387; doi:10.1186/s12918-017-0494-1)
Supplement: Additional file 1: — Additional information on methodology, statistical and computational analysis, supplemental figures. (PDF 6.66 mb) [file 12918_2017_494_MOESM1_ESM.pdf]

Table of Contents

1. LIBRARY SIZE **2**

2. EXPERIMENTAL PROCEDURE **2**

3. PRELIMINARY DATA ANALYSIS **3**

3.1 LINEAR RELATIONSHIP FSC CELL SIZE 3

3.2 VARIATION IN BIOLOGICAL REPLICATES 3

3.2 STRAIN BIAS IN KEIO COLLECTION ..... **ERROR! BOOKMARK NOT DEFINED.**

3.3 DEFINITION OF EXPRESSION VARIABLES 3

4. EXTREME VALUES AND CLUSTERS **4**

4.1 DEFINITION 4

4.2 DISTRIBUTION OF EXTREME-VALUE GENES ACROSS THE KEIO COLLECTION 4

5. STATISTICAL ANALYSIS **5**

5.1 BOOTSTRAP 5

5.2 GENE ONTOLOGIES AND HEAT MAPS 5

SUPPLEMENTARY FIGURES **6**

REFERENCES **17**

1. Library size

The total number of KEIO strains transformed with the plasmid pEZ8-123 carrying constitutively expressed mVenus and mCherry was 3,835. Two different wild type clones were also added during data collection (see below). The whole KEIO collection library had ~745 unlabeled wells, which contained viable *E. coli* cells and were not included in the final dataset. Also, according to our determination of the targeted genes we found 55 duplicated knockouts, bringing the number of unique genes to 3780 including wild type.

2. Experimental procedure

All data were acquired with a Guava Flow Cytometer (FC) as follows: 24-36 strain cultivations at a time (2-3 rows for each 96 well plate) were inoculated at a 1:80 dilution from overnight-cultures in warm MOPS rich medium supplemented with 0.5% glucose, and grown for exactly 1h15', in a shaker incubator at 37C. At the end of the incubation, the OD of the culture was read with the help of a microtiter plate reader to determine the number of cultures at mid-exponential phase (a 0.3-1.0 range corresponded to a spectrophotometer OD reading of ~1.3-2.0). These cultures were diluted 1:200 in PBS + translation inhibitor, and single-cell readings acquired via flow cytometry. The Spearman correlation between the log phase OD at sample collection, fluorescence and the other cell measures was between -0.01 and 0.03.

Two cell populations could be observed in the FC data in the SSC/FSC signal plots: one population with a higher average SSC and one with a lower SSC, both populations showed an almost identical average FSC. Because of the higher sensitivity of SSC for cells shape, we reasoned that the two populations with different SSC signals represent doubling and non-doubling cells. Normally the lower population was found to be about four times the size of the upper population. The total number of cells gathered for follow-up analysis was about 2000-3000 cells per sample.

Cell population centroids were generated, which determined population cell density and drew a Region Of Interest (ROI) around the cells to separate them from cell debris and instrument noise.

3. Preliminary data analysis

3.1 Linear relationship FSC cell size

We used different sized polypropylene fluorescence and size calibration beads (Amersham) to determine the correlation between the measured SSC and FSC channel values and the effective particle size. We found very good, linear correspondence between the measures and in particular that of the Forward Scatter, with the effective particle diameter within the range of common *E. coli* cell volumes (0.5 – 1.5 μ, Fig. S1).

3.2 Variation in biological replicates

The systematic and random error associated with plate-wise measurement was tested with technical replicates of 180 strains from three different plates and collected in 4 different days, and was found to be $5*10^{-4}$ for mVenus and $1.3*10^{-3}$ for mCherry. These values were approximately 1 order of magnitude smaller than the variance across all KEIO strains for the measured variables, which was between $3.6*10^{-3}$ and $1*10^{-2}$, excluding a substantial experimental error in the dataset.

3.3 Definition of expression variables

The impact of genotypic context on synthetic gene expression output was quantified by first eliminating the variation in mCherry and mVenus fluorescence across the whole dataset due to variation in cell size, as measured by the value of FSC defining the variable **S** (size). The fluorescence measure was regressed against the FSC measure with the R function 'lm' and the value of fluorescence predicted from the fit was found with the 'predict' function of the basic R package. The unexplained portions (residuals) were calculated by subtracting the value predicted from the original values, obtaining the values $\mathbf{mC^{reg}}$ and $\mathbf{mV^{reg}}$.

The set of values obtained represent the impact of the gene deletion on reporter gene expression from the synthetic genetic probe normalized for the effect on cell volume. The average between $\mathbf{mC^{reg}}$ and $\mathbf{mV^{reg}}$ was used throughout the manuscript as measure **E** for expression.

To calculate the differential effect of the knockout on the fluorescence output of each of the two reporter genes, $\mathbf{mC^{reg}}$ and $\mathbf{mV^{reg}}$ were further regressed against the variable **E** and the residuals were calculated as above from the fit. The correlation between the two new set of residuals from the second fit and **E** was very high (respectively 0.93 and 0.98), as it was the correlation between the two regressed values (0.84).

Significant residuals in this new measure (the two fluorescence values have identical absolute value but opposite sign), were identified as knockouts with a significant gene-specific divergence ($\mathbf{G_{spec}}$ measure).

4. Extreme values and clusters

4.1 Definition

For each of the **S**, **E** and **G** value distributions we selected the top and bottom 5% of the values as extremes, thus selecting 190 upper and lower **S** and **E** values, and 384 in total with a $\mathbf{G_{spec}}$ phenotype (Figs. S3-6).

4.2 Distribution of extreme-value genes across the KEIO collection

We determined whether the functionally enriched sets of genes in the phenotypic patterns of the cell-expression context showed any enrichment in specific plates. We used R for plotting the distribution of **S** or **E** variables of the whole dataset, and then plotted the position of the identified extreme genes.

Extreme **S** upper values were enriched in KEIO plates #23 to #47, whereas the extreme lower values spanned the whole collection (Fig. S7). The distribution of **E** outliers seemed to span the whole KEIO plate range with 3-5 hot-spots around plate #43-47 for upper extreme values and #57-59 for lower extreme values (Fig. S8). The distribution of $\mathbf{G_{spec}}$ outliers spanned the KEIO plates, with two hot-spots associated with lower extreme values centered on plate #63 and plate #95 (Fig. S9). It did not appear that there was a relationship between strain hot-spots across the KEIO plates.

Co-localization of strains with similar **S** or **E** phenotype in same plates was observed: genes with a $\mathbf{S_{high}}$ phenotype were concentrated in the range between plate #17 and #43 (Fig. S7), several $\mathbf{E_{high}}$ genes were found in plates #33, #37 and #39. There did not appear to be a clear link between plate co-localization and extreme **S** – **E** values. In particular the genes with a $\mathbf{S_{high}}$ phenotype including various aromatic amino acid biosynthetic genes (Group 1, main table 1) just 4/12 of the genes were located in plate #41 (Fig. S10 panel A). Genes with a $\mathbf{E_{high}}$ pattern, either in isolation or associated with a $\mathbf{S_{high}}$ pattern, were scattered across the dataset (Fig. S10 panel B), as were genes with a $\mathbf{S_{low}}$ pattern (Fig. S10 panel C). Genes with a $\mathbf{E_{low}}$ phenotype were found in several KEIO plates (Fig. S10 panel D).

However, we found a relative presence of two functional groups associated with KEIO plate #45: the KEGG pathway flagellar assembly (**GO:0006935)** and several protein chaperons (**GO:0006457) (**Fig. S10 panel D, dark-pink dots). Among 21 of these genes, 15 were located on plate #45 while the others were from other KEIO plates. This plate also contained 3 of 8 genes involved in ECA biosynthesis (Group 16), which also presented a $\mathbf{E_{low}}$ phenotype (Fig. S10 panel D, green dots). These genes shared a $\mathbf{E_{low}}$ phenotype also together with 7 other chemotactic/motility genes and proteases (ClpP/X) that resided in different KEIO plates.

5. Statistical analysis

5.1 Bootstrap

P-values through bootstrapping was implemented in R by using a simple sampling and hypothesis testing algorithm. In general, a n=10000 random sampling with replacement was applied to form groups of genes of the same size of the different GO groups discussed in the main text having data in the study. The null hypothesis was that the number of genes with a given phenotype (for example a $\mathbf{S_{high}}$-$\mathbf{E_{high}}$ phenotype) in the GO groups discussed in this study is the same as for groups formed with random genes across the dataset.

5.2 Gene Ontologies and heat maps

Gene Ontology biological classes were extracted from EcoCyc [1], where parent and child classes were mined for gene members. Functional enrichment of GO classes and KEGG pathways [2] was performed with the online DAVID bioinformatic resources version 6.8 [3]. A cutoff of the Bonferroni-corrected p-value was automatically applied by the online resource and corresponded to $p < 0.5$.

Heat maps with the set of GO genes were drawn with R packages ggplot and heatmap.2 by using Z-normalization (mean-centered St. Dev.-fold) of the values and using a color range applied to the whole dataset while plotting the specific set of genes (Fig. S11).

Supplementary Figures

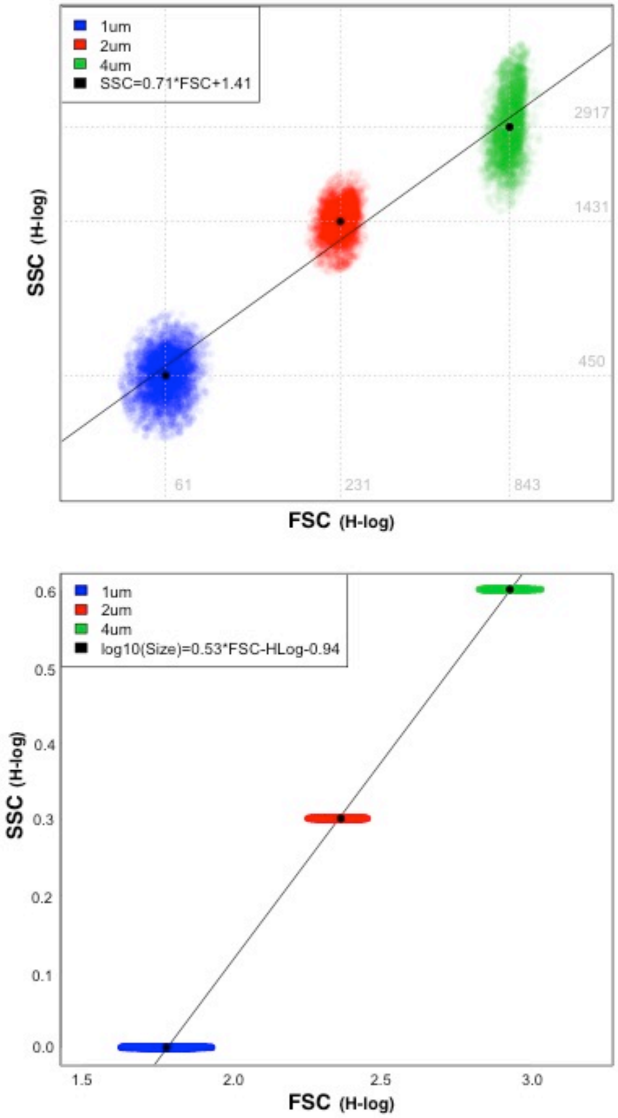

Fig. S1. Side Scatter (SSC) and Forward Scatter (FSC) measured for three particle sizes (A) and a linear regression of the measurements (B).

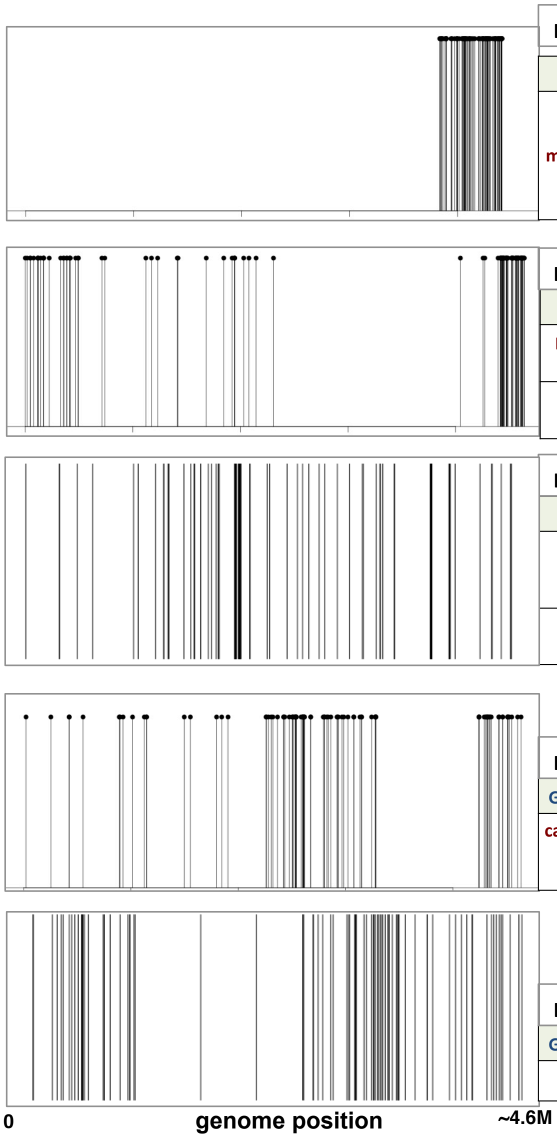

plate 37

GO pathway	n. genes
plasma membrane /cell wall	*adiC, actP, fieF, cbrB, yidG, yidH, yidI, yihN, yijD, yjcH, yjdF, yjeH, yjeO, yjdL, ompL, yihG, hsrA, psiE, yjbE, purP, yjcD, setC, yfjL, yjcE, yjbH, yjeP, yjeT*

plate 39

GO pathway	n. genes
DNA metabolic process	*mutL, polA, holC, holD, dinF, hsdM, mrr, priB, hsdM, hsdS, uvrA*
cell wall	*aroP, brnQ, cynX, codB, betT, fhuB, metN, mdlA, araJ, seqA, hofC, tauC*

plate 45

GO pathway	n. genes
flagellar assembly	*fliF, flhA, flhB, fliP, fliQ, fliT, fliD, fliK, motA, motB, fliM, fliN, fliG, fliS, fliI, flhC*
chemotaxis	*cheR, motA, motB, fliM, fliN, fliG, trg, cheW*

plate 57

GO pathway	n. genes
carbohydrate catabolic process	*kbaY, kbaZ, srlB, exuT, fucP, cmtB, ascF, cmtA, ptsP, gudP*

plate 61

GO pathway	n. genes
ribosome	*rplK, rpmE, rpmG, rplI*

Fig. S2. Left panels: genome position of genes in the indicated plates. Right panels: DAVID GO enrichment among position-associated genes (brackets). Plates were selected for having FSC and/or mCherry average median greater than 2 St. Dev. from the median of all plates.

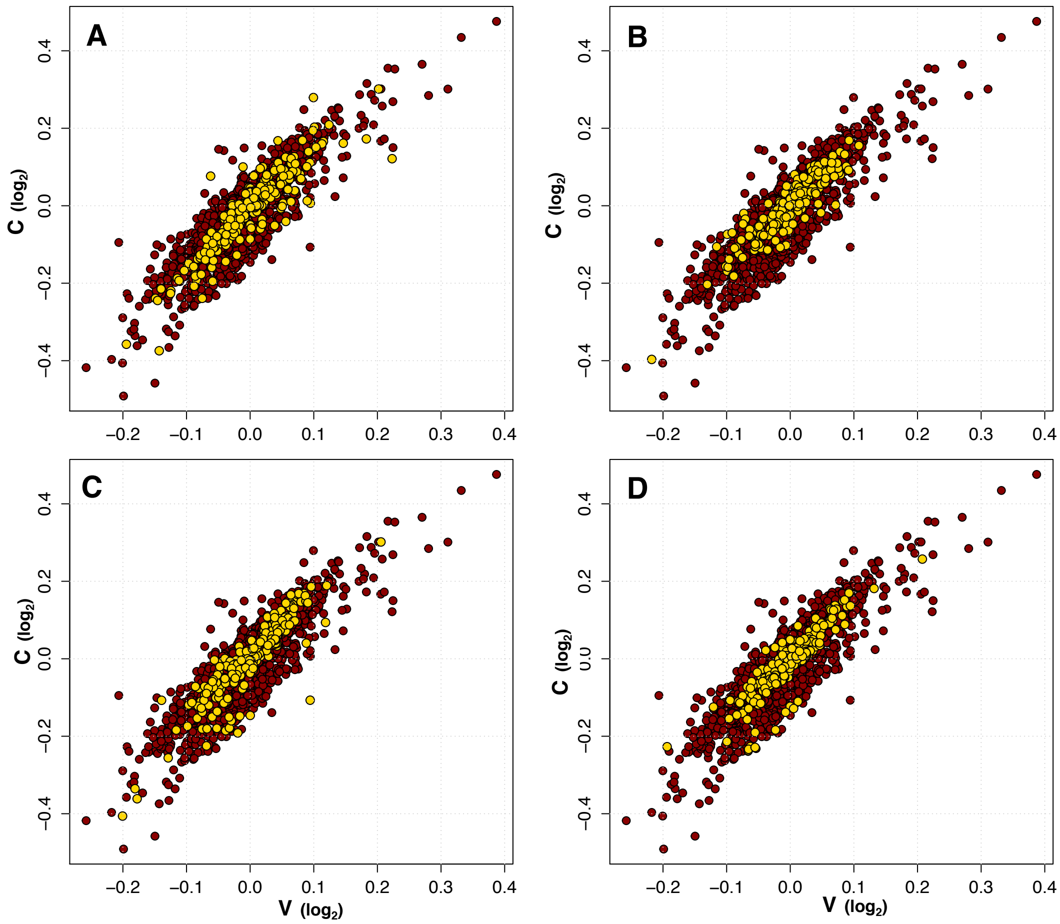

Fig. S3. Average mCherry (C) and mVenus (V) distribution for subsets of plates (gold) compared to all strains (maroon). In particular: plates 1-5 **(A)**, plates 7-11 (**B**), plates 13-17 **(C)** and plates 19-23 (**D**).

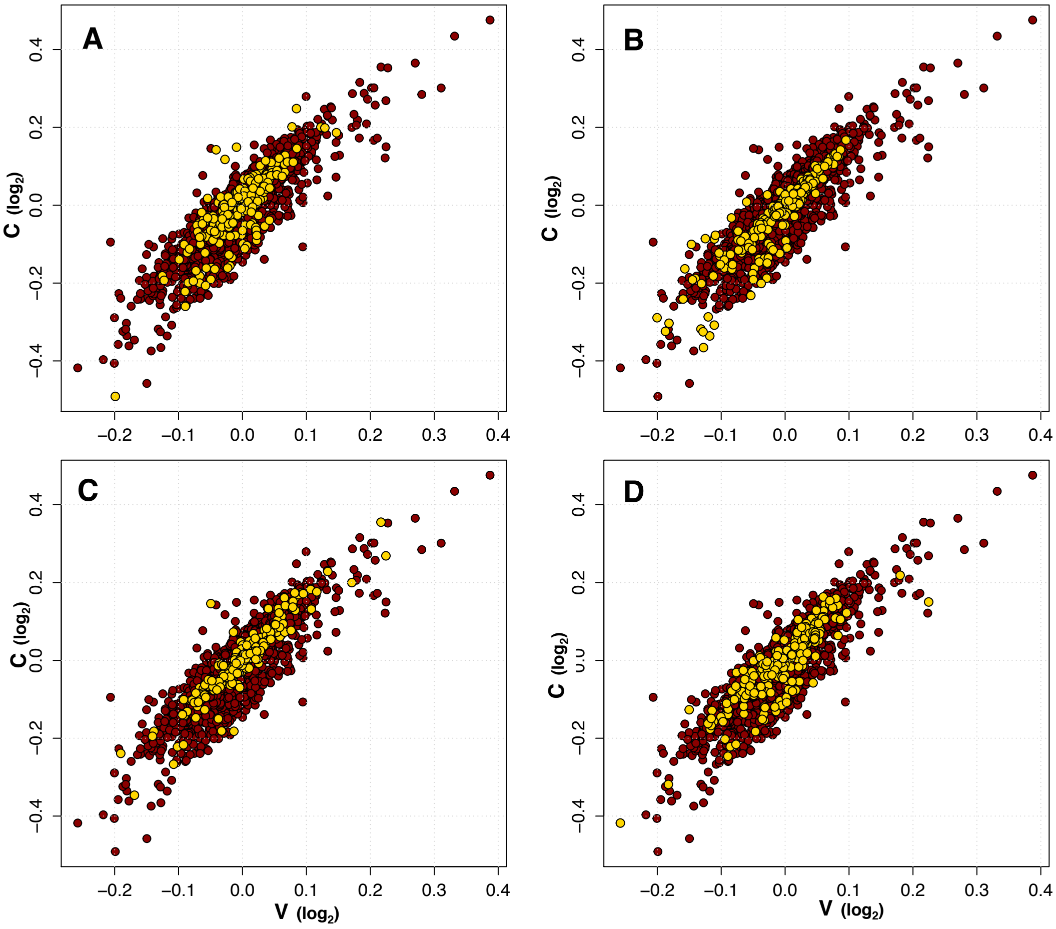

Fig. S4. Average mCherry (C) and mVenus (V) distribution for subsets of plates (gold) compared to all strains (maroon). In particular: plates 25-29 **(A),** plates 31-35 (**B**), plates 37-41 (**C)** and plates 43-47 (**D**).

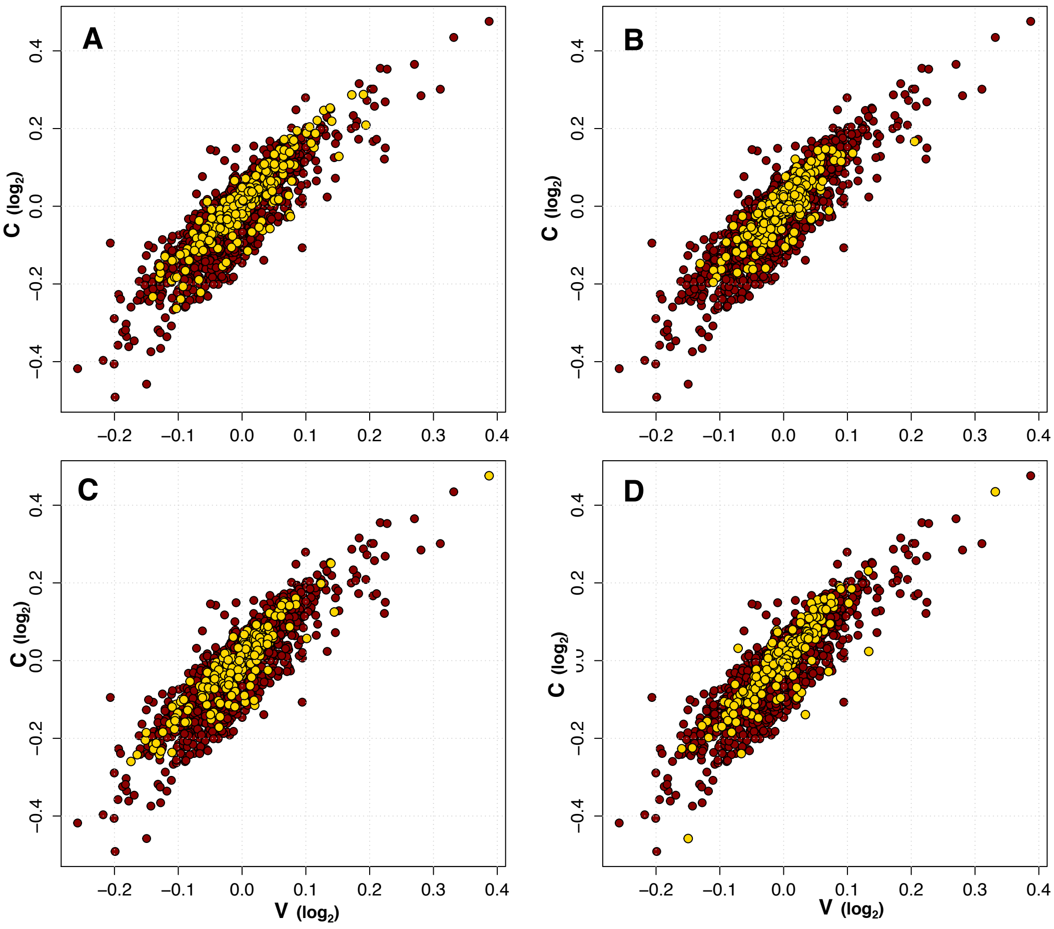

Fig. S5. Average mCherry (C) and mVenus (V) distribution for subsets of plates (gold) compared to all strains (maroon). In particular: plates 49-53 **(A)**, plates 55-59 (**B**), plates 61-65 (**C)** and plates 67-71 (**D**).

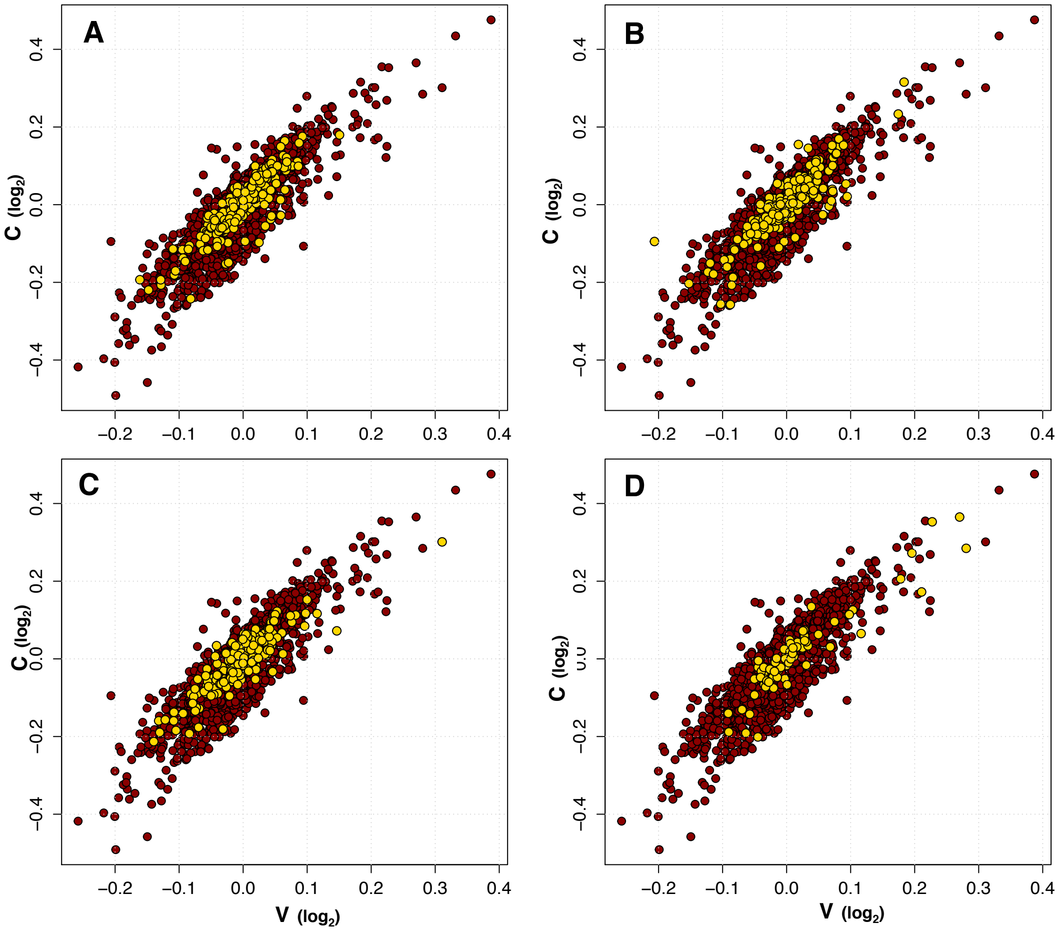

Fig. S6. Average mCherry (C) and mVenus (V) distribution for subsets of plates (gold) compared to all strains (maroon). In particular: plates 73-77 **(A)**, plates 79-83 (**B**), plates 85-89 (**C)** and plates 91-95 (**D**).

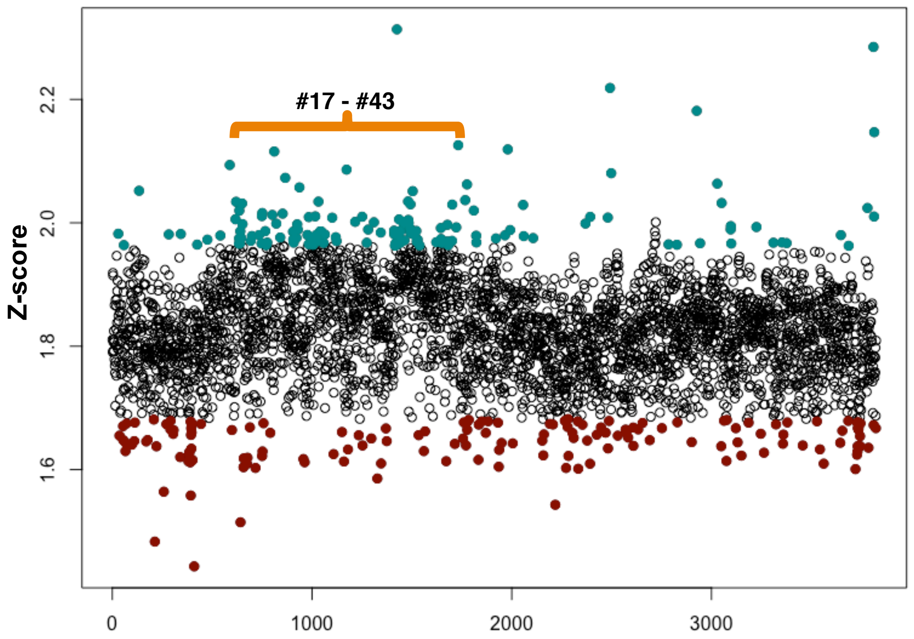

Fig. S7. Distribution of **S** values measured across the KEIO collection (cyan: values in top 5%, red: values in bottom 5%) (Z-score of regressed flow cytometer measurements, see Sup. Methods for more details).

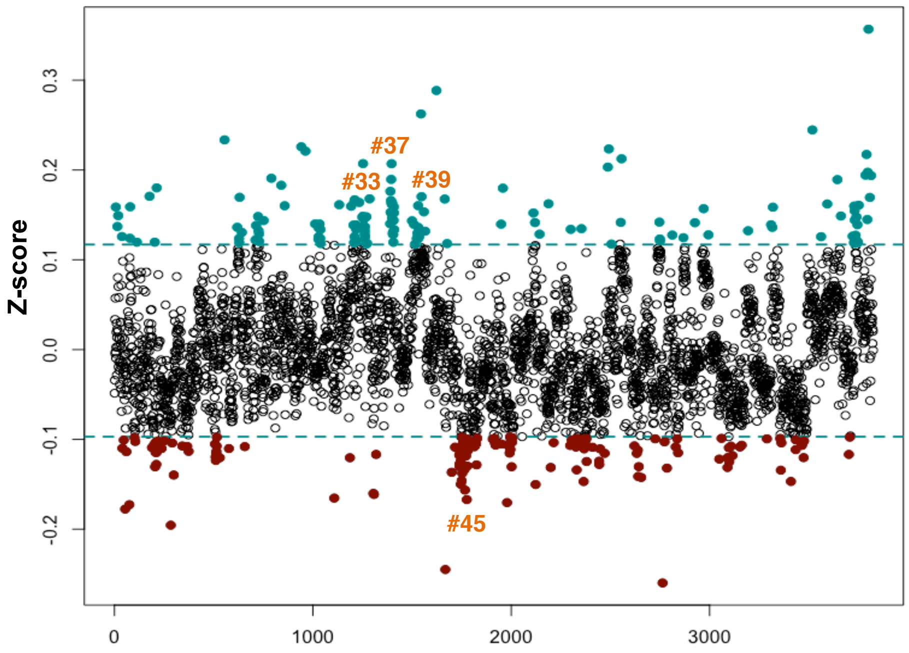

Fig. S8. Distribution of **E** values measured across the KEIO collection (cyan: values in top 5%, red: values in bottom 5%) (Z-score of regressed flow cytometer measurements, see Sup. Methods for more details).

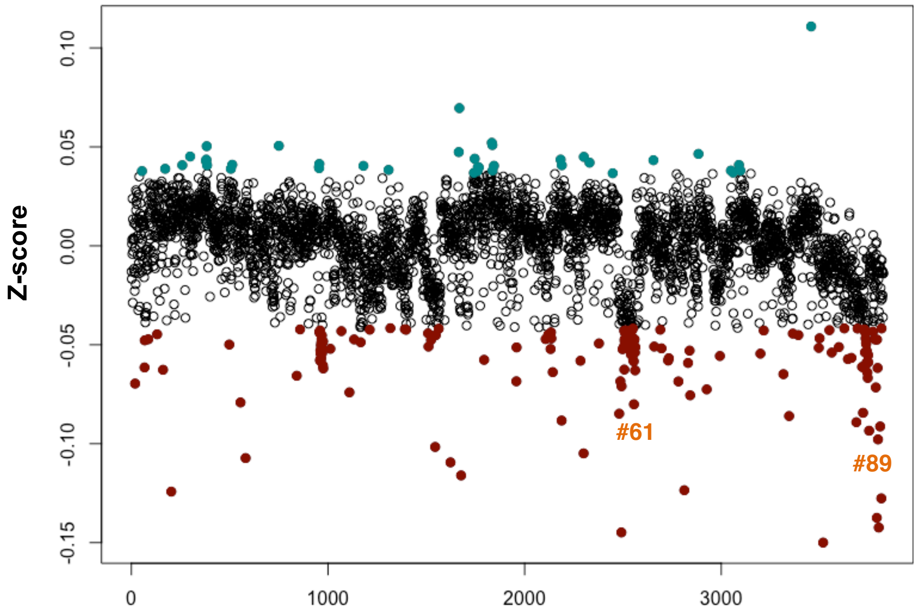

Fig. S9. Distribution of $\mathbf{G_{spec}}$ values measured across the KEIO collection (cyan: values in top 5%, red: values in bottom 5%) (Z-score of regressed flow cytometer measurements, see Sup. Methods for more details).

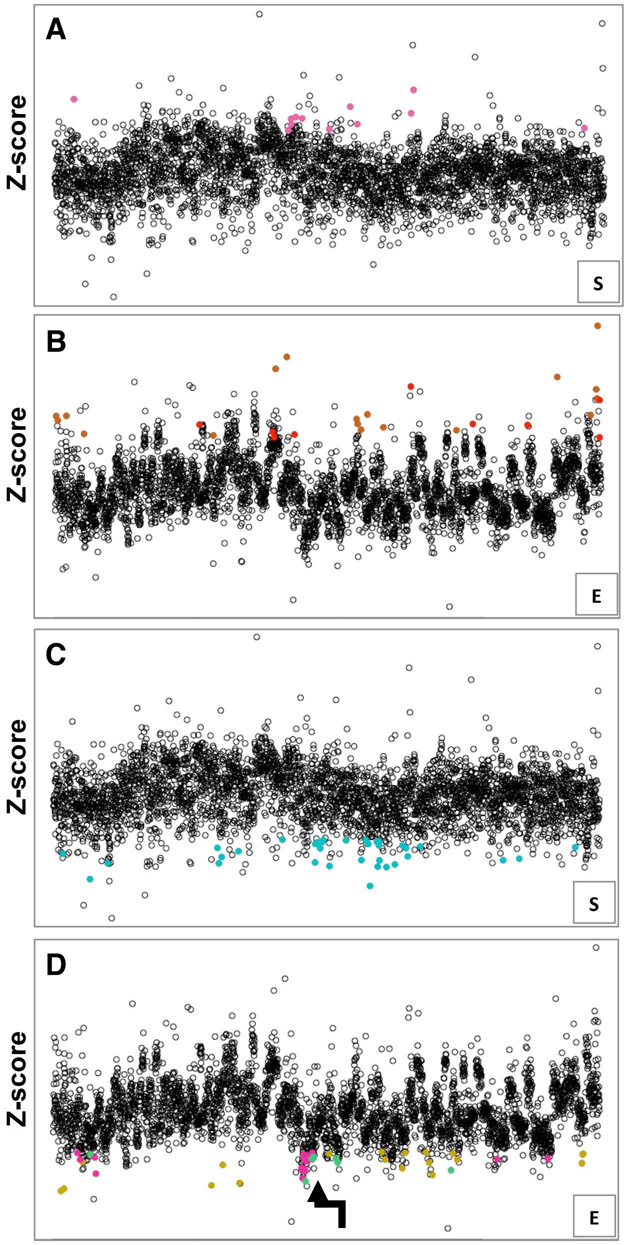

Fig. S10. Distribution of genes with significant **S** or **E** values enriched in specific phenotypic groups (main Fig. 3). Specifically, genes with an exclusively $\mathbf{S_{high}}$ phenotype (A, dark-pink dots), with either an exclusive $\mathbf{E_{high}}$ or combined $\mathbf{E_{high}}$-$\mathbf{S_{high}}$ phenotype (B, respectively dark orange and red dots), exclusive $\mathbf{S_{low}}$ phenotype (C, dark-cyan dots), or genes with a $\mathbf{E_{low}}$ variable alone or in combination (D, dark gold) including just the motility and chaperone functions (main group 15, D dark-pink) and the ECA synthesis function (group 16, D, dark green).

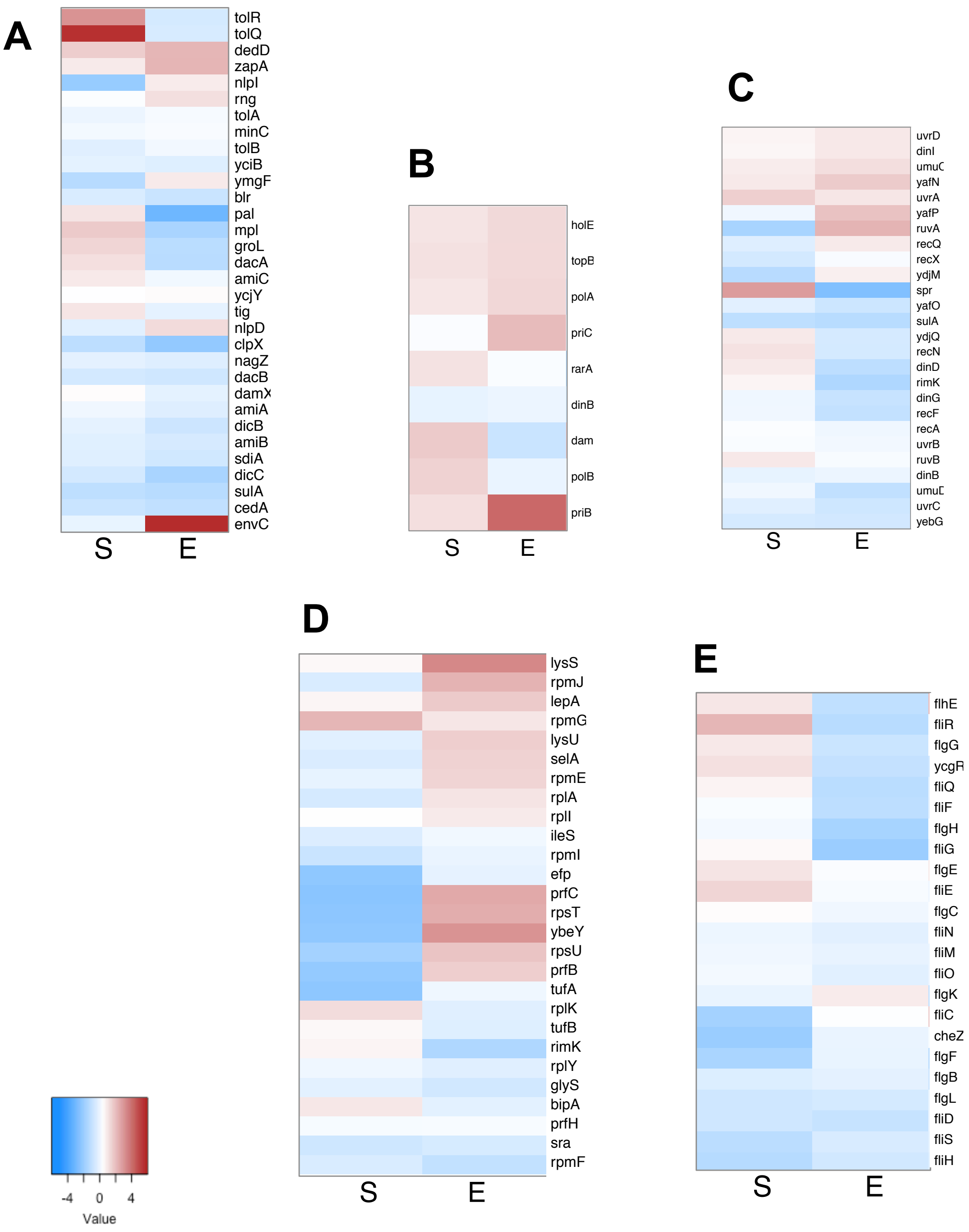

Fig. S11. Heat map of the **S** and **E** features associated with knockouts of genes involved in bacterial cell division (**A** ,GO:0051301), bacterial DNA replication (**B**, GO:0006261), bacterial DNA repair (**C**), cellular translation (**D**, GO:0006412). bacterial-type flagellum (**E**, GO:0009288).

References

1. Keseler IM, Bonavides-Martínez C, Collado-Vides J, Gama-Castro S, Gunsalus RP, Johnson DA, et al. EcoCyc: a comprehensive view of Escherichia coli biology. Nucleic Acids Res. 2009;37:D464–70.

2. Wixon J, Kell D. The Kyoto encyclopedia of genes and genomes--KEGG. Yeast. 2000;17:48–55.

3. Huang DW, Sherman BT, Lempicki RA. Systematic and integrative analysis of large gene lists using DAVID bioinformatics resources. Nat. Protoc. 2008;
